# Exploration of Blood Metabolite Signatures of Colorectal Cancer and Polyposis through Integrated Statistical and Network Analysis

**DOI:** 10.3390/metabo13020296

**Published:** 2023-02-17

**Authors:** Francesca Di Cesare, Alessia Vignoli, Claudio Luchinat, Leonardo Tenori, Edoardo Saccenti

**Affiliations:** 1 Magnetic Resonance Center (CERM), University of Florence, 50019 Sesto Fiorentino, Italy; 2 Department of Chemistry “Ugo Schiff”, University of Florence, 50019 Sesto Fiorentino, Italy; 3 Consorzio Interuniversitario Risonanze Magnetiche di Metallo Proteine (C.I.R.M.M.P.), 50019 Sesto Fiorentino, Italy; 4 Laboratory of Systems and Synthetic Biology, Wageningen University & Research, 6708 WE Wageningen, The Netherlands

**Keywords:** metabolomics, colorectal cancer, polyposis, network inference, mass spectrometry, multivariate data exploration, cancer metabolism

## Abstract

Colorectal cancer (CRC), one of the most prevalent and deadly cancers worldwide, generally evolves from adenomatous polyps. The understanding of the molecular mechanisms underlying this pathological evolution is crucial for diagnostic and prognostic purposes. Integrative systems biology approaches offer an optimal point of view to analyze CRC and patients with polyposis. The present study analyzed the association networks constructed from a publicly available array of 113 serum metabolites measured on a cohort of 234 subjects from three groups (66 CRC patients, 76 patients with polyposis, and 92 healthy controls), which concentrations were obtained via targeted liquid chromatography-tandem mass spectrometry. In terms of architecture, topology, and connectivity, the metabolite-metabolite association network of CRC patients appears to be completely different with respect to patients with polyposis and healthy controls. The most relevant nodes in the CRC network are those related to energy metabolism. Interestingly, phenylalanine, tyrosine, and tryptophan metabolism are found to be involved in both CRC and polyposis. Our results demonstrate that the characterization of metabolite–metabolite association networks is a promising and powerful tool to investigate molecular aspects of CRC.

## 1. Introduction

Colorectal cancer (CRC) is the third most commonly diagnosed cancer and the second leading cause of cancer-related death worldwide [1]. The major risk factors for developing CRC include older age, male sex, inflammatory bowel disease, alcohol intake, smoking, obesity, and family history [2,3]. Early-stage diagnosis is associated with a good prognosis (~90% 5-year survival), however, survival declines substantially when the tumor is identified at advanced stages [4]. In its early stages, CRC remains often asymptomatic; thus, organized screening programs aimed at increasing CRC early diagnosis are needed to decrease its morbidity and mortality [3]. Currently, occult blood in feces is the most commonly used screening analysis in clinical practice; however, it lacks the sensitivity and specificity needed for unambiguous early diagnosis [5]. Colonoscopy is recognized as the gold-standard diagnostic method for CRC: it offers high sensitivity, specificity, and accuracy; unfortunately, it is a costly and extremely invasive procedure [6]. Therefore, the development of new methodologies able to identify CRC with minimal invasiveness and high accuracy at an early stage is highly desirable.

It is well known from the clinical literature that about 95% of CRC begin as colonic adenomatous polyps [7]. A series of still not completely characterized molecular alterations induce CRC growth. Broadly speaking, the development of CRC can be attributable to a complex multistep process, ignited by the growth of a benign polyp with the potential to evolve into an in-situ carcinoma by the accumulation of additional somatic mutations [8,9]. When detected, polyps’ excision and adequate treatment [10] can prevent further tumor development. To develop therapeutic strategies able to prevent the transition from health epithelium to polyps to CRC it is necessary to fully understand the puzzle of the underlying biochemical mechanisms. The biochemistry of early carcinogenesis is controlled by an interplay between genomic susceptibility, metabolic reprogramming, and the local microenvironment. The development of adenomatous polyps probably passes through the impact of oxidative stress on the metabolic pathways involved in the renewal of the colon epithelium. The immune system also plays a role because chronic inflammation promotes cell proliferation and differentiation. Last, but not least, microbiota dysregulation may influence microenvironmental homeostasis altering the integrity of the colon mucosa [8].

Considering that all these entangled effects have an impact on the local and systemic patient metabolism, metabolomics represents a valid instrument to provide further insights into the CRC metabolic mechanisms. Metabolomics has already proved to be an excellent tool for biomedical investigations [11,12,13,14], and it has been opportunely applied to the study of CRC adopting different analytical strategies and kinds of samples [5,15]. To expand the vision from the particular to the general, it is necessary to perform a further step considering the shape of the metabolite-metabolite association networks [16]. Changes in metabolite association patterns are associated with changes in the pathophysiological conditions, and the resulting networks can be compared across conditions under the postulate that the differences and commonalities observed in the reconstructed networks faithfully mirror the reshape of the underlying biological networks [17,18,19,20].

**Figure 1:**
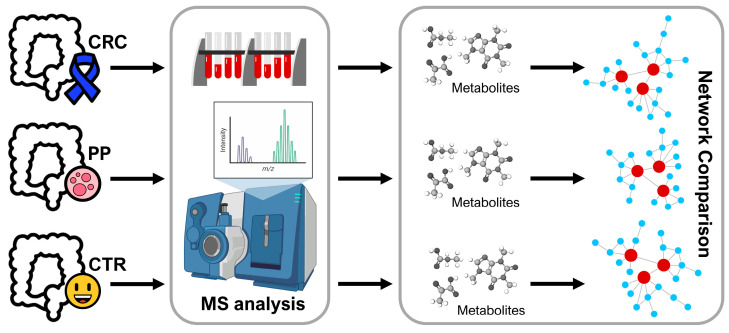
Study design of our study on colorectal cancer (CRC) patients, polyposis (PP) patients and healthy controls (CTR).

With this premise, a publicly available dataset published by Zhu et al. [21] was reanalyzed applying a network approach (Figure 1).

In the original study, the authors applied a mass spectrometry-based metabolic profiling approach to discover candidate biomarkers for CRC detection using human serum samples. The authors found a robust metabolic signature able to discriminate healthy controls (CTR), CRC, and polyp patients (PP). From our analysis, it emerges that the network calculated on serum metabolites of CRC patients has a different architecture with respect to that of PP and CTR, while no relevant differences emerged comparing colon and rectus cancers.

## 2. Material and Methods

### 2.1. Dataset Description

We re-analyzed the metabolomics data set collected by Zhu et al. [21]. Data and sample information were downloaded from the UC San-Diego Metabolomics Workbench public repository (https://www.metabolomicsworkbench.org/ (accessed on 23 June 2021) with the following Project ID number PR000226. For further details on sample collection and preparation, Mass Spectrometry (MS) experiments, and metabolite annotation and quantification we refer the readers to the original paper and the associated supplementary material [21].

The original data set contains 113 metabolites measured using MS on *n* = 234 serum samples from the three different groups. They are abundances of MS signals, obtained by peak integrals and reported in counts per second. Patients with colorectal cancer (CRC) which included patients with colon and rectal cancers, patients with polyposis (PP), and healthy controls (CTR) were enrolled, and were age- and gender-matched in each group.

We removed *n* = 8 outliers (see Section 2.2.1), leaving *n* = 226 samples/subjects for analysis, (Figure S1) divided into *n* = 65 CRC patients (*n*_1_ = 35 men, *n*_2_ = 30 women), *n* = 74 PP patients (*n*_1_ = 35 men, *n*_2_ = 39 women), and *n* = 87 CTR (*n*_1_ = 42 men, *n*_2_ = 45 women).

The mean age ± standard deviation (SD) of each group is 58.4 ± 13.3 years, 55.5 ± 7.0 years, and 54.2 ± 13.6 years, for CRC, PP, and CTR respectively. The *n* = 65 CRC patients were also divided into *n* = 39 colon cancer patients (*n*_1_ = 20 men, *n*_2_ = 19 women) and *n* = 26 rectal cancer patients (*n*_1_ = 15 men, *n*_2_ = 11 women), with a mean of age ± SD of 58.7 ± 14.2 years and 58.0 ± 12.0 years, respectively.

### 2.2. Data Pre-Processing

#### 2.2.1. Data Overview and Normalization

Outliers were determined as those samples/subjects falling outside the 95% confidence ellipses on a two-dimensional space reduced with Principal Component Analysis (PCA); a total of 8 patients (3.4%) (1 CRC patient, forming part of the rectal cancer sub-group, 2 PP patients, and 5 CTR) were removed from the analysis (Figure S1).

Metabolites’ abundances were normalized using “RankNorm” function before analysis. The offset *k* = 3/8 default parameter, corresponding to Blom’s transformation [22], was used. This method applies the rank-based inverse normal transform (INT) into two-steps. Firstly, the observations were transformed into the scale of probabilities using the empirical cumulative distribution function [23]. Subsequently, the observations were transformed into Z-scores, using the probit function [24].

### 2.3. Univariate Analysis

Variables were transformed by taking the square root of the values to correct for heteroscedasticity [25]. Univariate Student’s *t*-test [26] was used to compare normalized metabolite concentrations between patient groups. The Benjamini-Hochberg method was used to correct for multiple testing [27] and FDR adjusted *p*-values < 0.05 were considered statistically significant.

### 2.4. Multivariate Analysis

Principal Component Analysis [28,29] was applied to data scaled to unit variance to explore data patterns. The Random Forest (RF) algorithm [30,31,32,33] was employed for pairwise classification comparing CRC vs. CTR, CRC vs. PP, and PP vs. CTR. To reduce the potential bias due to an unbalanced number of subjects/samples per group, *k* = 100 resampling was imposed, retaining 85% of data for each group to be compared. Accuracy, sensitivity, specificity, the area under the ROC curve (AUROC), and corresponding 95% CIs were calculated according to the standard definitions and given as average over the 100 re-samplings. The significance of the models was determined with a permutation-test using *n* = 1000 permutations [34].

### 2.5. Network Analysis

#### 2.5.1. Reconstruction of Metabolite-Metabolite Association Network

The Probabilistic Context Likelihood of Relatedness based on Correlation (PCLRC) [35] algorithm was used to infer metabolite-metabolite association networks. To remove non-significant background correlations, this algorithm provides a robust evaluation of the correlation using a resampling strategy in combination with the previously published Context Likelihood of Relatedness [36] approach. Spearman correlation was used as a measure of association between metabolites. The PCLRC algorithm outputs a probability matrix P giving the likelihod *p_ij_* for each Spearman correlation *r_ij_* between metabolites *i* and *j*. A detailed description of the PCLRC approach is provided in the Supplementary Methods (Section 1). The method is well suited to infer association networks when the correlation is used as a measure of association [37].

#### 2.5.2. Pathway Enrichment Analysis

Pathway enrichment analysis on the set of statistically differential connected metabolites obtained comparing CRC vs. CTR and PP vs. CTR was performed using the tool available on MetaboAnalyst 5.0 (www.metaboanalyst.ca (accessed on 30 January 2023), implementing a hypergeometric test. As reference (universe) metabolome all compounds in the selected pathway library, (KEGG *Homo sapiens*) were used [38,39]. The pathway impact score is calculated by this tool as the sum of the importance measures of the matched metabolites normalized by the sum of the importance measures of all metabolites in each pathway. Only enriched pathways with an impact score > 0.01 were considered.

#### 2.5.3. Measures for Network Topology

Network topology and node (metabolite) characteristics were evaluated using several standard metrics in addition to connectivity (node degree). Average shortest path length, betweenness centrality, closeness centrality, clustering coefficient, degree, eccentricity, neighborhood connectivity, radiality, stress, and topological coefficient, calculated using Network Analyzer [40], a Java plugin available for the Cytoscape platform (https://cytoscape.org (accessed on 21 July 2021) were used [41]. A brief overview of the measures is given in the Supplementary Methods (Section 2).

### 2.6. Software

Calculations were performed using R statistical software (version 3.3.2). The R implementation of the PCRLC algorithm is available at the following link: semantics.systemsbiology.nl. The RNOmi R package [42] was used to normalize metabolic abundance. The “randomForest” function from the R Random Forest library [43] was used to generate RF-classification models, using default parameters. Permutation tests were performed using the “rp.importance” function, from the R rfPermute library. The network visualization, the estimation of network topology, and the network statistics were performed using Cytoscape platform [41] (version 3.8.2), integrated with the NetworkAnalyzer plugin [40].

## 3. Results

### 3.1. Exploratory Analysis of Serum Metabolomic Profiles of CRC, Polyposis, and Healthy Patients

The PCA score plot (Figure S2a) shows that the CRC, PP patients, and CTR subjects are not separated, suggesting that metabolic differences are too subtle to be resolved using an unsupervised multivariate approach.

To evaluate the metabolic differences among the three groups, a univariate Student’s *t*-test was used. As reported in Table S1, 24 metabolites showed statistically significant (FDR adjusted *p*-value < 0.05) differences comparing CRC with CTR subjects. Instead, no significantly different metabolite was found comparing PP with CTR subjects. 23 metabolites showed statistically significant (FDR adjusted *p*-value < 0.05) differences comparing CRC with PP subjects (Table S1).

### 3.2. Classification of CRC, PP, and CTR

We used Random Forest classification to investigate whether the metabolomic profiles could be used to discriminate in a predictive way CRC, PP, and CTR subjects (Table 1). Overall, good classification models to discriminate between CRC and CTR (78.2% mean accuracy) (Figure S2b) and between CRC and PP patients (79.5% mean accuracy) (Figure S2c) were obtained. A weaker predictive model (62.2% mean accuracy) (Figure S2d) was obtained for the discrimination of CTR and PP. Hippuric acid, malonic acid/3-hydroxybutyric acid, linolenic acid, histidine, glycochenodeoxycholate, adenylsuccinate, phosphoenolpyruvic acid (PEP), glyceraldehyde, fructose 1,6-bisphosphate/fructose-2,6 bisphosphate (F16BP/F26BP), linolenic acid, maleic acid, adipic acid, glycocholate and gamma-aminobutyrate are the most relevant and significant variables in the model discriminating CRC with respect to CTR (Figure S3a). PEP, adenosine, glyceraldehyde, methionine, hippuric acid, 2-deoxyuridine, linolenic acid, creatinine, xanthurenate, linoleic acid, uridine, glycocholate, aspartic acid, glycochenodeoxycholate, dimethylglycine, adipic acid, glutaric acid, lysine, and histidine are the metabolites that contribute significantly to the discrimination between CRC and PP (Figure S3b). Only few variables, namely F16BP/F26BP, adenosine, tryptophan, xanthurenate, salicylurate, G16BP, oxaloacetate, glyceraldehyde, and histidine, significantly discriminate PP and CTR subjects. Taken together these results indicate the presence of a strong metabolic signature specific to CRC patients and of a weaker signature specific to polyposis (Figure S3c).

### 3.3. Analysis of Metabolite-Metabolite Association Networks Specific to CRC, PP, and CTR

**Figure 2:**
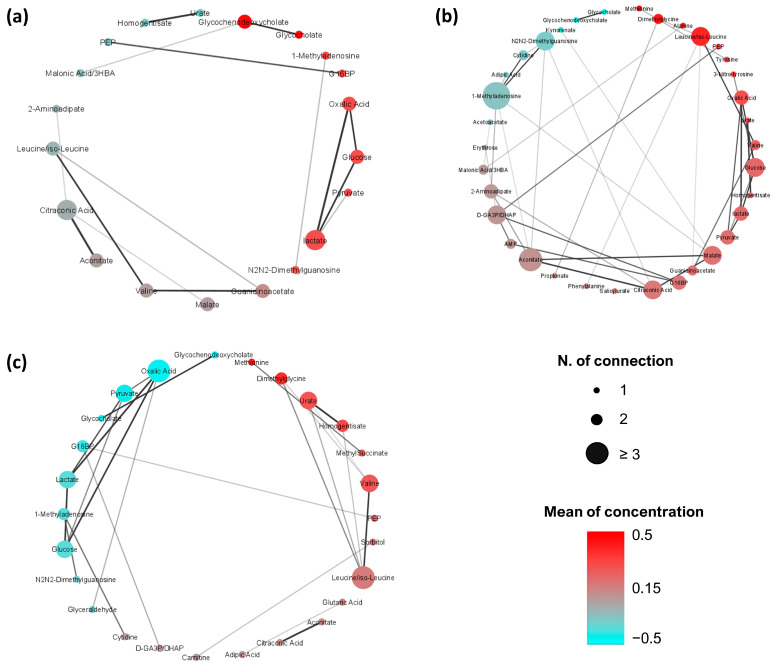
Metabolite-metabolite association network of (**a**) CRC patients; (**b**) PP patients; (**c**) CTR. Only the significantly (Benjamini-Hochberg adjusted *p*-value ≤ 0.05) different connections among metabolites are reported. Nodes are colored according to their mean of concentration (from light blue to red) and their dimension is proportional to the increasing metabolite-metabolite degree of connectivity. Edges represent correlation with |*r*| ≥ 0.6 and their transparency depends on the likelihood of the metabolic connections.

The metabolite-metabolite association networks of CRC, PP, and CTR are shown in Figure 2. Comparing the three metabolic structures, it emerged that the CRC-specific network has a different topology than PP and CTR networks. In particular, the CRC network (Figure 2a) tends to be poorly connected but establishing robust connections (|*r_ij_*| > 0.6) between glucose, lactate, oxalic acid, aconitate, citraconic acid, leucine/isoleucine, valine, and guanidinoacetate. In contrast, the PP (Figure 2b) and the CTR networks (Figure 2c) are more interconnected, showing very similar topologies.

### 3.4. Comparison of the Topological Properties of the Metabolite-Metabolite Association Network

**Figure 3:**
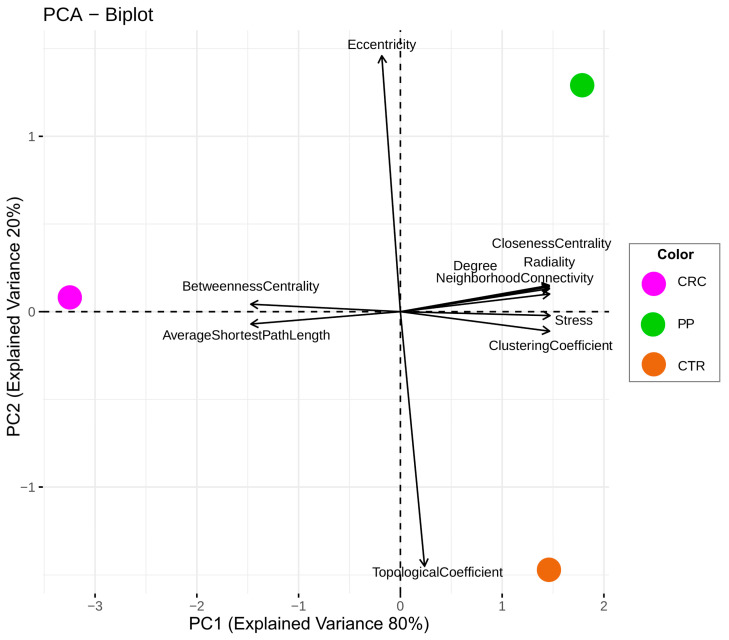
Principal component analysis (PCA) biplot performed on topological metabolite-metabolite association network parameters. PCA dots are colored according to the CRC (magenta dot), PP (green dot), and CTR (dark orange dot) groups. PCA loadings represent the following network statistical parameters: average shortest path length, betweenness centrality, closeness centrality, clustering coefficient, degree, eccentricity, neighborhood connectivity, radiality, stress, and topological coefficient.

To compare comprehensively CRC, PP, and CTR metabolite-metabolite association networks, the topological and statistical network parameters via PCA (Figure 3) were examined. Analyzing the PCA biplot, the network specific to PP and CTR subjects are characterized by different topological coefficient and eccentricity. This means that the nodes in the two networks tend to have different shared neighbors. Eccentricity is a measure of centrality, thus indicating the importance of a node (i.e., metabolite) in the networks, and in this case, the two networks tend to have different important nodes. The CRC network differs from the PP and CTR networks by node characteristics like betweenness, centrality, and average shortest path length, which describe how the different nodes are interconnected, reflecting the low connectedness of the CRC network. Moreover, the metabolite-metabolite association networks for the two CRC sub-groups (colon and rectal cancer) show no differences in terms of topological parameters (Figure 3).

### 3.5. Differential Connectivity Analysis

**Figure 4:**
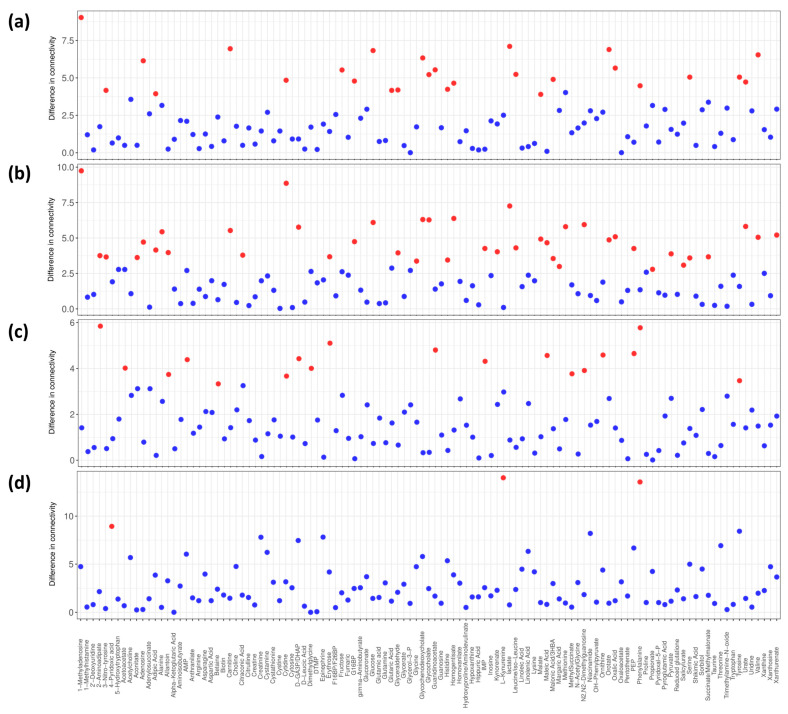
Differential connectivity analysis. Differences in terms of connectivity in metabolite-metabolite association networks of (**a**) CRC and CTR groups; (**b**) CRC and PP groups; (**c**) PP and CTR groups; (**d**) colon cancer and rectal cancer sub-groups. Blue dots correspond to a not statistically significant metabolite and red dots correspond to a statistically significant metabolite. The threshold for significance of FDR corrected *p*-values ≤ 0.05 was imposed.

To quantify the difference in metabolite association patterns and to highlight possible underlying metabolic differences within the three groups as well as within the two CRC sub-groups (colon and rectal cancers), differential connectivity analysis was performed comparing CRC and PP networks against the CTR network, the CRC against PP, and the colon against rectal cancers (Figure 4 and Supplementary Table S2).

We observed several metabolites that are differentially connected in the CRC and CTR networks (1-methyladenosine, 3-nitrotyrosine, adenosine, adipic acid, carnitine, cytidine, fructose, glucose 1,6-bisphosphate, glucose, glutaric acid, glyceraldehyde, glycochenodeoxycholate, glycocholate, guanidinoacetate, histidine, homogentisate, lactate, leucine/isoleucine, malate, malonic acid/3HBA (3-Hydroxybutyric acid), orotate, oxalic acid, phenylalanine, serine, tyrosine, urate, valine) (Figure 4a) and CRC and PP networks (1-methyladenosine, 2-aminoadipate, 3-nitrotyrosine, aconitate, adenosine, adipic acid, alanine, allantoin, carnitine, citraconic acid, cytidine, D-GA3P (D-Glyceraldehyde 3-phosphate)/DHAP (Dihydroxyacetone phosphate), erythrose, glucose 1,6-bisphosphate, glucose, glyceraldehyde, glycine, glycochenodeoxycholate, glycocholate, histidine, homogentisate, IMP (Inosine monophosphate), kynorenate, lactate, leucine/isoleucine, malate, maleic acid, malonic acid/3HBA, margaric acid, methionine, N2,N2-dimethylguanosine, orotate, oxalic acid, PEP, propionate, pyruvate, salicylurate, serine, succinate/methylmalonate, urate, valine, xanthurenate) (Figure 4b). In contrast, a limited number of differentially connected metabolites (2-aminoadipate, acetoacetate, allantoin, AMP, betaine, cytidine, D-GA3P/DHAP, dimethylglycine, erythrose, guanidinoacetate, IMP, maleic acid, methylsuccinate, N2,N2-dimethylguanosine, ornithine, PEP, phenylalanine, tyrosine) was observed when comparing the CTR and PP networks (Figure 4c), in complementary agreement with the Random Forest predictive models (Table 1). When comparing the networks of the two sub-groups of colon and rectal cancers, only for 4-pyridoxic acid, L-kynurenine, and phenylalanine (Figure 4d) showed significantly different connections, which indicates similarity between the two networks.

**Figure 5:**
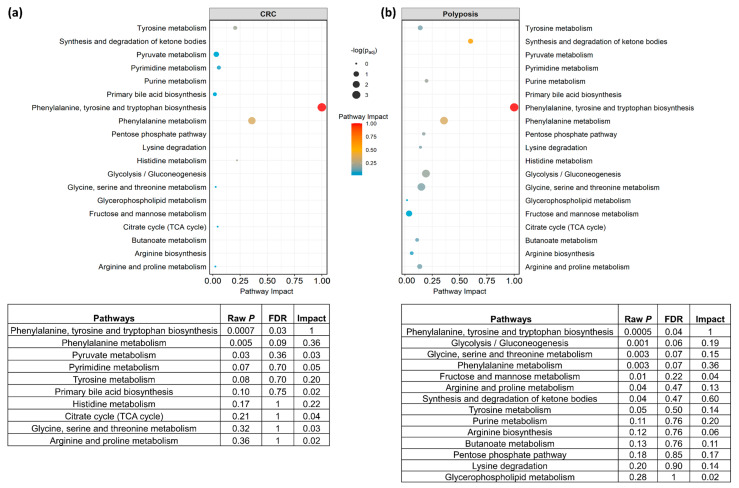
Pathway analysis plot according to the metabolites presenting significantly (Benjamini-Hochberg adjusted *p*-value ≤ 0.05) different connections in (**a**) CRC compared with CTR, and (**b**) PP compared with CTR. Dots are colored according to the metabolic pathway impact score and their dimensions are proportional to their significance. Only enriched pathways with an impact score per cohort > 0.01 were considered. For each pathway, raw *p*-value, FDR adjusted *p*-value, and the impact score are also reported.

Metabolite pathway enrichment analysis was performed on the metabolites which had shown significant (Benjamini-Hochberg adjusted *p*-value ≤ 0.05) different connections comparing CRC vs. CTR, and PP vs. CTR (Figure 5). A total of 14 unique enriched pathways (impact score > 0.01) in CRC compared with CTR network, and in PP compared with CTR network was found. The most impacted (impact score > 0.15) and differential enriched pathways are: (i) the phenylalanine, tyrosine, and tryptophan biosynthesis and phenylalanine metabolism in both CRC and PP as compared to CTR, (ii) the synthesis and degradation of ketone bodies in PP as compared to CTR, and (iii) the glycolysis/gluconeogenesis and the glycine, serine, and threonine metabolism in PP as compared to CTR. The only metabolic pathway statistically significant after FDR correction in both comparisons is the phenylalanine, tyrosine, and tryptophan biosynthesis.

## 4. Discussion

Colorectal carcinomas principally evolve from adenomatous polyps. Fortunately, more than 90% of adenomas do not progress to cancer; however, it is currently not possible to reliably identify those that will progress and those that will not, thus the complete resection of polyps during colonoscopy represents the only available option to eliminate the risk of cancer derived from those adenomas [44]. The peculiar metabolic alterations as well as the individual immune-metabolic response induced by the presence of cancer constitute the characteristic metabolic signature of CRC patients [5,21]. In these analyses, we present an evaluation of the serum metabolomic profiles of CRC and PP patients with respect to control subjects using a metabolite–metabolite association networks approach to investigate the existence of metabolic molecular mechanisms underlying these different clinical conditions.

Supervised balanced RF models show good discriminations for the comparisons between CRC and CTR and between CRC and PP obtaining an AUC of 0.875 and 0.871 respectively. Conversely, PP and CTR present only slight differences (AUC of 0.661). Despite the different statistical approaches employed, our results are in line with those reported in the original publication of Zhu [21] and in another publication focused on a subset of this population [45]. These data confirm that CRC patients develop systemic metabolic alterations that are not present, not only in CTR but also in PP patients.

We compared the metabolite-metabolite association networks of CRC, PP, and CTR to explore the magnitude, topology, and architecture of metabolite connections and their variability. It emerges that the network calculated on serum samples of CRC patients has an architecture completely different from the ones of PP and CTR, whereas no relevant difference emerges comparing colon and rectus cancers.

The CRC network is characterized by a relatively low number of strong connections. This difference is corroborated also by the differential connectivity analysis and by the analysis of the topological parameters: CRC differs from PP and CTR by betweenness, centrality, average shortest path length, and the number of differential connections, reflecting the lower interconnection of this network. We can speculate that the defragmentation of the CRC network could be the result of the multiple activations of several metabolic pathways and that these profound alterations are reflected at the systemic level in sera of CRC patients. Of note, nodes present in the CRC network are all directly or indirectly related to the different pathways involved in the energetic metabolism. Cancer cells need to meet a high energy demand to support cell proliferation and migration, thus they acquire molecular substrates and energy through unusual metabolic pathways. This need induces a profound rewiring of their metabolic network that extends beyond the Warburg effect and alterations of individual metabolic fluxes [46,47,48]. Considering the number of differentially connected metabolites, the CRC network showed 27 and 42 statistically different connections (22 are in common) when compared to CTR and PP networks, respectively (Figure 4a,b), and the differences are mainly associated with phenylalanine, tyrosine, and tryptophan biosynthesis, phenylalanine metabolism, and pyruvate metabolism.

The landscape of metabolic alterations associated with polyposis appears to be more heterogenous than the ones associated with colorectal cancer. PP and CTR are poorly discriminated and appear to have similar network architectures with more interconnections. However, they are characterized by different topological coefficient and eccentricity; this means that the same node (metabolite) in the two networks has different importance and is connected to different metabolites. A panel of 18 metabolites (Figure 4c) shows to have significantly (Benjamini-Hochberg adjusted *p*-value ≤ 0.05) different connections in the association networks of PP and CTR. The pathways associated with phenylalanine, tyrosine and tryptophan biosynthesis, phenylalanine metabolism, glycolysis/gluconeogenesis, glycine, serine and threonine metabolism, fructose and mannose metabolism, arginine and proline metabolism, and synthesis and degradation of ketone bodies show to be the most affected by these connectivity variations. Interestingly, the phenylalanine, tyrosine, and tryptophan metabolism reveal to be the only significant pathway in both CRC and PP. Tryptophan and phenylalanine are essential amino acids: tryptophan metabolism is linked to the production of serotonin while phenylalanine is required to produce tyrosine, which is catalyzed by phenylalanine hydroxylase. It has been shown that phenylalanine hydroxylase activity can be altered in inflammation or malignancy [49,50] and some metabolomic studies, using different methodological approaches, corroborate its alteration in CRC [48,51,52,53]. Tyrosine is converted into several metabolites, including L-DOPA, pyruvate, fumarate, and phenol, the latter conversion is mediated by the enzyme tyrosine phenol-lyase (β-tyrosinase) [54]. Alterations in the profiles of these metabolites have been reported in colorectal cancer [55] and other cancer types [56,57] but, to the best of our knowledge, alterations in these pathways have never been reported in association with polyposis.

## 5. Conclusions

We reanalyzed a publicly available data set [21] of blood metabolites of patients with colorectal cancer, polyposis, and healthy controls. We expanded the original analysis integrating classical univariate analysis of metabolite abundances with predictive multivariate modelling and with the analysis of metabolite-metabolite association patterns. From the present analyses emerged that both CRC and polyposis possess specific metabolic signatures that distinguish them from healthy control profiles. In both cases, significant deregulation of phenylalanine, tyrosine, and tryptophan metabolism were observed. However, the metabolic landscape associated with polyposis appears to be more variegated than that of colorectal cancer, with several affected pathways, although these dysregulations are subtle.

## Figures and Tables

**Table 1:** Mean values of accuracy, specificity, sensitivity and area under the curve (AUC) of RF models built comparing CRC vs. CTR patients, CRC vs. PP patients, and PP vs. CTR patients.

	Mean Accuracy % (95% CI, *p*-Value)	Mean Specificity % (95% CI, *p*-Value)	Mean Sensitivity % (95% CI, *p*-Value)	AUC (95% CI, *p*-Value)
CRC vs. CTR	78.2 (77.8–78.5, 0.001)	79.0 (78.6–79.3, 0.001)	77.1 (76.6–77.7, 0.001)	0.875 (0.873–0.877, 0.001)
CRC vs. PP	79.5 (79.2–79.9, 0.001)	77.7 (77.1–78.3, 0.002)	81.2 (70.8–81.7, 0.003)	0.871 (0.869–0.872, 0.001)
PP vs. CTR	62.2 (61.8–62.6, 0.004)	63.1 (62.3–63.8, 0.001)	61.5 (60.9–63.8, 0.012)	0.661 (0.658–0.665, 0.004)

## Data Availability

Data were retrieved from the Metabolomics Workbench public repository with the following Project ID number PR000226 (https://www.metabolomicsworkbench.org/ (accessed on 23 June 2021)).

## Supplementary Materials

The following supporting information can be downloaded at: https://www.mdpi.com/article/10.3390/metabo13020296/s1, Table S1: Statistically significant (FDR adjusted *p*-value < 0.05) metabolic differences obtained using univariate Student’s *t*-test comparing CRC vs. PP and CRC vs. CTR. The significant metabolites highlighted by Zhu et al. are indicated with the following symbol (*); Table S2: The statistical significance of the differentially connected metabolites comparing CRC vs. PP, CRC vs. CTR, PP vs. CTR, and colon cancer vs. rectal cancer networks; Figure S1: Principal Component Analysis (PCA) score plot; Figure S2: Principal Component Analysis model and Balanced Random Forest score plots; Figure S3: Metabolite importance calculated by Mean Decrease Gini score for classification between: (a) CRC and CTR, (b) PP and CTR, and (c) CRC and PP; Figure S4: Topological network parameters of metabolite-metabolite association networks. Sorce: [27,35,36,40,41,58,59,60,61,62,63,64,65]
