# Supplementary material for: Exploration of Blood Metabolite Signatures of Colorectal Cancer and Polyposis through Integrated Statistical and Network Analysis
Source: Metabolites. 2023 Feb 17;13(2):296. doi: 10.3390/metabo13020296 (PMC9965766; doi:10.3390/metabo13020296)
Supplement: Supplementary file 1 [file metabolites-13-00296-s001.zip › metabolites-2194545-supplementary.pdf]

Exploration of blood metabolite signatures of colorectal cancer and polyposis through integrated statistical and network analysis

Francesca Di Cesare, Alessia Vignoli, Claudio Luchinat, Leonardo Tenori and Edoardo Saccenti

SUPPLEMENTARY METHODS

1. *Reconstruction of metabolite association network*

The Probabilistic Context Likelihood of Relatedness based on Correlation (PCLRC)[1] algorithm was used to infer metabolite-metabolite association networks. In order to remove non-significant background correlations, this algorithm provides a robust evaluation of the correlation using a resampling strategy in combination with the previously published Context Likelihood of Relatedness (CLR)[2] approach. The PCLRC algorithm gives like output a probability matrix **P** showing the likelihood p_{ij} for each revealed Spearman correlation r_{ij} between two metabolites *i* and *j*. We considered correlation for which the probabilistic value p_{ij} was > 97% and we set to 0 all remaining correlations:

$$r_{ij} = \begin{cases} r_{ij} & if p_{ij} \geq 0.97 \\ 0 & if p_{ij} < 0.97 \end{cases} \tag{2}$$

The 0.97 probability threshold was chosen as the best compromise between network complexity (*i.e.* number of nodes) and interpretability. PCRLC was used with default parameters, with 1000 resampling iterations, and 75% of samples kept in each iteration and the top 30% correlation retained for each iteration.

2.2.1. Differential connectivity analysis

Given a specific network *a*, the connectivity χ_i^a for each metabolite *i* is described as:

$$\chi_i^a = \left(\sum_{j=1}^{J} |r_{ij}| \right) - 1 \tag{3}$$

Moreover, the differential connectivity $\Delta_i^{a,b}$ for each metabolite *i* among two networks *a* and *b* is calculated as follow:

$$\Delta_i^{a,b} = \chi_i^a - \chi_i^b \quad (4)$$

The statistical significance of the differentially connected metabolites was determined by means of a permutation-test. In order to eliminate the relationship between variables and in order to maintain their variance, the columns of each input matrices were independently permuted defining a permutated matrix $\mathbf{X}_{(k)}$. The overall network estimation is performed on permutated data matrix, generating the related Spearman correlation $R_{(k)}$ analysis. These estimations were subsequentially used to assess, for each metabolite contained in the permutated matrix $\mathbf{X}_{(k)}$, the permutated connectivity (Equation (4)), and the permutated differential connectivity (Equation (5)):

$$\chi_{i,k}^a = \left(\sum_{j=1}^{J} \left|r_{ij}^k\right|\right) - 1 \quad (5)$$

$$\Delta_{i,k}^{a,b} = \chi_{i,k}^a - \chi_{i,k}^b \quad (6)$$

The permutation step was repeated k = 1000 yielding a null distribution D_i of permutated differential connectivity values. The significance of a given differential connectivity value $\Delta_i^{a,b}$ (estimated from the non-permutated original data) was calculated as a *P*-value, according to the following formula:

$$P - value = \frac{1 + \left(|D_i| > \left|\Delta_i^{a,b}\right|\right)}{k} \quad (7)$$

All *P*-values were corrected for multiple test comparisons using the Benjamini-Hochberg approach[3].

2. Measures for network topology

Network topology and node (metabolite) characteristics were evaluated using several standard metrics in addition to connectivity (node degree). We used *Average Shortest Path Length*, *Betweenness Centrality*, *Closeness Centrality*, *Clustering Coefficient*, *Degree*, *Eccentricity*, *Neighborhood Connectivity*, *Radiality*, *Stress*, and *Topological Coefficient*. These measures were calculated using Network Analyzer[4], a Java plugin available for the Cytoscape platform[5]. A brief overview of the measure is given in the following.

The *Average Shortest Path Length* [6] , also known as the characteristic path length, indicates the expected distance between two connected nodes. The shortest path length is considered the shortest distance between two nodes *i* and *j*, denoted by L(*i*,*j*). The shortest path length distribution gives the number of node pairs (*i*,*j*) with L(*i*,*j*). = *k* for *k* = 1,2, …, and it indicates small-word properties of a network.

The *Eccentricity* of a metabolite *a* is the maximum noninfinite length of the shortest path between a metabolite *i* and another metabolite in the network.

The *Betweenness Centrality* Bc_i [7] of a node *i* is calculated as follows:

$$Bc_i = \sum_{s \neq i \neq j,} \frac{\sigma_{sij}}{\sigma_{sj}} \tag{8}$$

where *s* and *j* are nodes in the network different from node *i*, σ_{sj} denotes the number of shortest paths from nodes *s* and *j*, and σ_{sij} is considered the number of shortest paths from s to j passing through node *i*. The Betweenness Centrality could be normalized by dividing the number of node pairs excluding *i*:

$$\frac{Bc_i}{\frac{(N-1)(N-2)}{2}} \tag{9}$$

where N is the total number of nodes in the connected component that *i* belongs to. This parameter reflects the degree of control that given node exercises over the interactions of the other nodes in the network.

The *Closeness Centrality* Cc_i [8] of a node *i* is the reciprocal of the average shortest path length and it is a number between 0 and 1. Cc_i measures the distance of a node *i* to all other nodes and it is calculated as follows:

$$Cc_i = \frac{1}{\frac{\sum_j d_{ij}}{N-1}} \tag{10}$$

where d_{ij} is the distance between node *i* and *j*.

In the undirected networks, the *Clustering* C_i[6,9,10] of a node *i* is defined as a ratio $\frac{N}{M}$, where N is the number of edges between the neighbors of *i*, and M the maximum number of edges that

could possibly exist between the neighbors of *i*. This coefficient is always a number between 0 and 1. The network clustering coefficient is the average of the clustering coefficients of all nodes in the network and it is used to highlight a modular organization of metabolic networks[10].

The *Neighborhood Connectivity*[11] of node *i* is the average connectivity of all neighbors of *i*.

The *Radiality*[9] is a node centrality index and gives high centralities to nodes that have a small distance to any other node in their reachable neighborhood compared to their diameter.

The *Stress*[12] of a node *i* is the number of shortest paths passing through *i*.

The *Topological Coefficient* T_i[13] of a node *i* with k_n as the number of neighbors of node *i* is calculated as follows:

$$T_i = \frac{average\big(J(i,j)\big)}{k_i} \tag{11}$$

where the value $J(i,j)$ is the number of neighbors shared between the nodes *i* and *j*. This coefficient indicates the tendency of the nodes in the network to have shared neighbors.

In order to compare the metabolite-metabolite association networks topology, we evaluate the average of node characteristics and connectivity among metabolite *i* and *j.*

SUPPLEMENTARY REFERENCES:

1. Suarez-Diez M, Saccenti E. Effects of Sample Size and Dimensionality on the Performance of Four Algorithms for Inference of Association Networks in Metabonomics. *J Proteome Res*. 2015;14(12):5119-5130. doi:10.1021/acs.jproteome.5b00344

2. Akhand MAH, Nandi RN, Amran SM, Murase K. Context likelihood of relatedness with maximal information coefficient for Gene Regulatory Network inference. In: *2015 18th International Conference on Computer and Information Technology (ICCIT)*. ; 2015:312-316. doi:10.1109/ICCITechn.2015.7488088

3. Benjamini Y, Hochberg Y. Controlling the False Discovery Rate: A Practical and Powerful Approach to Multiple Testing. *Journal of the Royal Statistical Society Series B (Methodological)*. 1995;57(1):289-300.

4. Doncheva NT, Assenov Y, Domingues FS, Albrecht M. Topological analysis and interactive visualization of biological networks and protein structures. *Nature Protocols*. 2012;7(4):670-685. doi:10.1038/nprot.2012.004

5. Shannon P, Markiel A, Ozier O, et al. Cytoscape: A Software Environment for Integrated Models of Biomolecular Interaction Networks. *Genome Res*. 2003;13(11):2498-2504. doi:10.1101/gr.1239303

6. Watts DJ, Strogatz SH. Collective dynamics of ‘small-world’ networks. *Nature*. 1998;393(6684):440-442. doi:10.1038/30918

7. Yoon J, Blumer A, Lee K. An algorithm for modularity analysis of directed and weighted biological networks based on edge-betweenness centrality. *Bioinformatics*. 2006;22(24):3106-3108. doi:10.1093/bioinformatics/btl533

8. Freeman LC. Centrality in social networks conceptual clarification. *Social Networks*. 1978;1(3):215-239. doi:10.1016/0378-8733(78)90021-7

9. Barabási A-L, Oltvai ZN. Network biology: understanding the cell’s functional organization. *Nature Reviews Genetics*. 2004;5(2):101-113. doi:10.1038/nrg1272

10. Ravasz E, Somera AL, Mongru DA, Oltvai ZN, Barabási A-L. Hierarchical Organization of Modularity in Metabolic Networks. *Science*. 2002;297(5586):1551-1555. doi:10.1126/science.1073374

11. Maslov S, Sneppen K. Specificity and stability in topology of protein networks. *Science*. 2002;296(5569):910-913. doi:10.1126/science.1065103

12. Brandes U. A faster algorithm for betweenness centrality. *The Journal of Mathematical Sociology*. 2001;25(2):163-177. doi:10.1080/0022250X.2001.9990249

13. Stelzl U, Worm U, Lalowski M, et al. A Human Protein-Protein Interaction Network: A Resource for Annotating the Proteome. *Cell*. 2005;122(6):957-968. doi:10.1016/j.cell.2005.08.029

SUPPLEMENTARY TABLE

Table S1: Statistically significant (FDR adjusted *p*-value < 0.05) metabolic differences obtained using univariate Student's t-test comparing CRC vs PP and CRC vs CTR. No significance (FDR adjusted *p*-value < 0.05) was observed comparing PP vs CTR. The significant metabolites highlighted by Zhu et al. are indicated with the following symbol (*)

Metabolite	CRC vs. PP	CRC vs. CTR
1-Methyladenosine	> 0.05	0.04 (*)
2-Kynurenine	> 0.05 (*)	> 0.05
2-Aminoadipate	> 0.05	> 0.05 (*)
2'-Deoxyuridine	> 0.05 (*)	0.003 (*)
2-Oxoglutarate	> 0.05 (*)	> 0.05 (*)
Acetylcholine	> 0.05 (*)	> 0.05
Adenosine	> 0.05 (*)	> 0.05
Adenylosuccinate	> 0.05 (*)	0.03 (*)
Alanine	0.03 (*)	> 0.05
Allantoin	> 0.05 (*)	0.005 (*)
Alpha-Ketoglutaric Acid	0.01	0.01
Arginine	> 0.05	> 0.05 (*)
Asparagine	> 0.05 (*)	> 0.05
Aspartic Acid	0.01 (*)	0.01 (*)
Biotin	0.01 (*)	> 0.05
Creatinine	> 0.05 (*)	> 0.05 (*)
Cystathionine	> 0.05	0.01 (*)
Dimethylglycine	0.002 (*)	0.01 (*)
Fumarate	> 0.05 (*)	> 0.05 (*)
gama-Aminobutyrate	0.02 (*)	> 0.05 (*)
Glucose	> 0.05	> 0.05 (*)
Glutamic acid	0.03 (*)	0.03 (*)
Glutamine	0.001 (*)	0.003 (*)
Glutaric acid	> 0.05 (*)	> 0.05
Glyceraldehyde	0.0003 (*)	0.002 (*)
Glycerol-3-P	> 0.05 (*)	> 0.05
Glycochenodeoxycholate	0.004 (*)	0.01 (*)
Glycocholate	0.01 (*)	0.02 (*)
Histidine	0.0002 (*)	0.00002 (*)
Hydroxyproline/Aminolevulinate	> 0.05 (*)	0.04 (*)
Hyppuric Acid	0.0009 (*)	0.004 (*)
Homogentisate	> 0.05 (*)	> 0.05 (*)
Kynorenate	> 0.05 (*)	0.01 (*)
Lactate	> 0.05	> 0.05 (*)
Leucic acid	> 0.05 (*)	> 0.05 (*)
Leucine	> 0.05 (*)	> 0.05

Linoleic Acid	0.01 (*)	> 0.05 (*)
Linolenic Acid	0.0004 (*)	0.002 (*)
Lysine	0.00007 (*)	0.0005 (*)
Maleic Acid	> 0.05 (*)	0.01 (*)
Malonic acid/3-hydroxybutyrate (3HBA)	> 0.05 (*)	> 0.05 (*)
Margaric Acid	0.04 (*)	> 0.05
Methionine	0.00007 (*)	0.0005 (*)
Methylsuccinate	> 0.05 (*)	> 0.05 (*)
N-AcetylGlycine	0.01 (*)	0.03 (*)
OH-phenylpyruvate	> 0.05	> 0.05 (*)
Orotate	> 0.05 (*)	> 0.05
Oxalic Acid	> 0.05	0.04 (*)
Oxaloacetate	> 0.05 (*)	> 0.05
Pentothenate	> 0.05 (*)	> 0.05
PEP	0.002 (*)	0.04 (*)
Proline	> 0.05	> 0.05 (*)
Pyruvate	> 0.05	> 0.05 (*)
Threonine	> 0.05 (*)	> 0.05
Trimethylamine-N-oxide	> 0.05 (*)	> 0.05
Tryptophan	> 0.05 (*)	> 0.05
Urate	0.04 (*)	> 0.05 (*)
Uridine	0.0008 (*)	> 0.05 (*)
Xanthurenate	0.02 (*)	> 0.05

Table S2: The statistical significance of the differentially connected metabolites comparing CRC vs. PP, CRC vs. CTR, PP vs. CTR, and colon cancer vs. rectal cancer networks.

**Differentially connected metabolites**	**CRC vs. PP Adjusted *P*-value**	**CRC vs. CTR Adjusted *P*-value**	**PP vs. CTR Adjusted *P*-value**	**Colon vs. Rectal cancer Adjusted *P*-value**
1-Methyladenosine	0.005	0.008	0.509	1.000
1-Methylhistamine	0.663	0.959	0.915	1.000
2-Aminoadipate	0.011	0.770	0.009	1.000
2'-Deoxyuridine	0.577	0.979	0.822	1.000
3-Nitro-tyrosine	0.011	0.027	0.854	1.000
4-Pyridoxic acid	0.267	0.974	0.701	0.038
5-Hydroxytryptophan	0.088	0.974	0.402	1.000
Acetoacetate	0.077	0.974	0.016	1.000
Acetylcholine	0.572	0.094	0.113	0.618
Aconitate	0.009	0.974	0.073	1.000
Adenosine	0.005	0.008	0.746	1.000
Adenylosuccinate	0.941	0.434	0.071	1.000

Adipic Acid	0.009	0.038	0.936	1.000
Alanine	0.005	0.230	0.190	1.000
Allantoin	0.013	0.974	0.023	1.000
Alpha-Ketoglutaric Acid	0.446	0.974	0.890	1.000
Aminoisobutyrate	0.869	0.609	0.427	1.000
AMP	0.089	0.609	0.009	0.365
Anthranilate	0.852	0.974	0.630	1.000
Arginine	0.461	0.974	0.509	1.000
Asparagine	0.663	0.950	0.287	1.000
Aspartic Acid	0.249	0.974	0.348	1.000
Betaine	0.757	0.526	0.027	1.000
Biotin	0.346	0.974	0.701	1.000
Carnitine	0.005	0.008	0.509	1.000
Choline	0.838	0.759	0.287	1.000
Citraconic Acid	0.011	0.974	0.093	1.000
Citrulline	0.900	0.787	0.427	1.000
Creatine	0.671	0.974	0.712	1.000
Creatinine	0.226	0.883	0.963	0.094
Cystamine	0.147	0.336	0.641	0.348
Cystathionine	0.473	0.974	0.427	1.000
Cystine	0.977	0.835	0.693	1.000
Cytidine	0.005	0.011	0.027	1.000
Cytosine	0.967	0.974	0.693	1.000
D-GA3P/DHAP	0.005	0.974	0.009	0.099
Dimethylglycine	0.099	0.780	0.016	1.000
D-Leucic Acid	0.832	0.974	0.777	1.000
DTMP	0.267	0.974	0.427	1.000
Epinephrine	0.226	0.715	0.963	0.099
Erythrose	0.011	0.862	0.009	1.000
F16BP/F26BP	0.626	0.418	0.531	1.000
Fructose	0.099	0.011	0.092	1.000
Fumaric	0.147	0.974	0.701	1.000
G16BP	0.005	0.011	0.967	1.000
gama-Aminobutyrate	0.455	0.536	0.693	1.000
Glucoronate	0.834	0.289	0.190	1.000
Glucose	0.005	0.008	0.777	1.000
Glutamic acid	0.852	0.974	0.402	1.000
Glutamine	0.852	0.974	0.774	1.000
Glutaric Acid	0.060	0.027	0.488	1.000
Glyceraldehyde	0.011	0.027	0.785	1.000
Glycerate	0.626	0.974	0.297	1.000
Glycerol-3-P	0.089	1.000	0.195	1.000
Glycine	0.029	0.770	0.427	1.000
Glycochenodeoxycholate	0.005	0.008	0.915	0.618
Glycocholate	0.005	0.008	0.915	1.000
Guanidinoacetate	0.446	0.008	0.009	1.000
Guanosine	0.346	0.787	0.693	1.000

Histidine	0.029	0.015	0.872	0.826
Homogentisate	0.005	0.008	0.548	1.000
Homovanilate	0.256	0.974	0.161	1.000
Hydroxyproline/Aminolevulinate	0.757	0.835	0.465	1.000
Hypoxanthine	0.352	0.974	0.693	1.000
Hyppuric Acid	0.896	0.974	0.963	1.000
IMP	0.005	0.974	0.009	1.000
Inosine	0.147	0.609	0.936	1.000
Kynorenate	0.013	0.715	0.194	1.000
lactate	0.005	0.008	0.740	1.000
Leucine/iso-Leucine	0.005	0.008	0.853	1.000
Linoleic Acid	0.391	0.974	0.701	1.000
Linolenic Acid	0.147	0.974	0.183	0.277
L-Kynurenine	0.966	0.455	0.081	0.038
Lysine	0.226	0.974	0.915	1.000
Malate	0.009	0.035	0.693	1.000
Maleic Acid	0.005	0.980	0.009	1.000
Malonic Acid/3HBA	0.013	0.011	0.547	1.000
Margaric Acid	0.047	0.294	0.872	1.000
Methionine	0.005	0.065	0.427	1.000
MethylSuccinate	0.346	0.937	0.009	1.000
N2,N2-Dimethylguanosine	0.005	0.682	0.009	1.000
N-AcetylGlycine	0.568	0.787	0.934	1.000
Niacinamide	0.626	0.320	0.509	0.057
OH-Phenylpyruvate	0.791	0.572	0.427	1.000
Ornithine	0.264	0.320	0.009	1.000
Orotate	0.005	0.008	0.126	1.000
Oxalic Acid	0.005	0.008	0.509	1.000
Oxaloacetate	0.791	1.000	0.742	1.000
Pentothenate	0.474	0.974	0.967	1.000
PEP	0.005	0.974	0.009	0.226
Phenylalanine	0.467	0.011	0.009	0.038
Proline	0.089	0.770	0.934	1.000
Propionate	0.048	0.251	0.994	1.000
Pyridoxal-5-P	0.564	0.974	0.895	1.000
Pyroglutamic Acid	0.626	0.294	0.402	1.000
Pyruvate	0.013	0.835	0.134	1.000
Reduced glutatione	0.587	0.950	0.936	1.000
Salicylurate	0.031	0.691	0.773	1.000
Serine	0.015	0.008	0.531	1.000
Shikimic Acid	0.626	0.974	0.693	1.000
Sorbitol	0.877	0.289	0.259	1.000
Succinate/Methylmalonate	0.013	0.121	0.915	1.000
Taurine	0.877	0.974	0.953	1.000
Threonine	0.352	0.937	0.804	0.226
Trimethylamine-N-oxide	0.928	0.253	0.099	1.000
Tryptophan	0.141	0.974	0.488	1.000

Tyrosine	0.398	0.008	0.031	0.068
Urate	0.005	0.011	0.521	1.000
Uridine	0.869	0.320	0.281	1.000
Valine	0.005	0.008	0.509	1.000
Xanthine	0.106	0.835	0.805	1.000
Xanthosine	0.626	0.974	0.488	1.000
Xanthurenate	0.005	0.289	0.381	1.000

SUPPLEMENTARY FIGURES

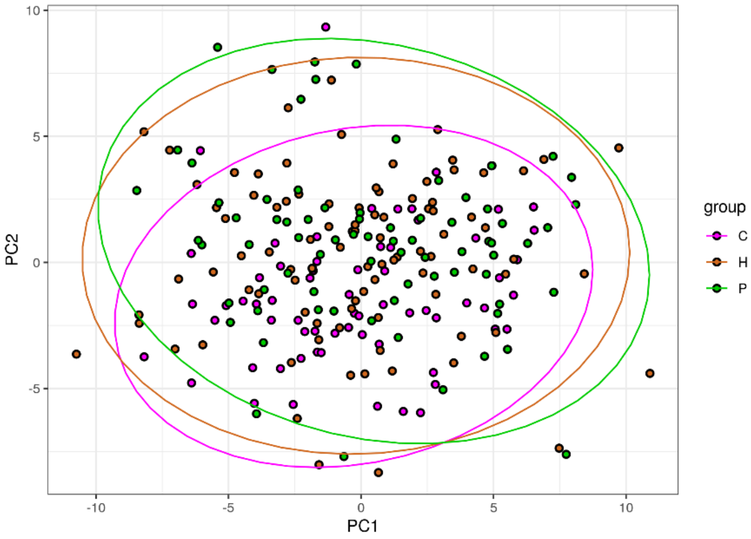

Figure S1: Principal Component Analysis (PCA) score plot. Each dot represents a single metabolic profile coloured by the different groups of patients: *n*=66 CRC patients (magenta dots), *n*=76 PP patients (green dots), and *n*=92 CTR patients (dark orange dots). For each group of patients, 95% confidence ellipses were reported.

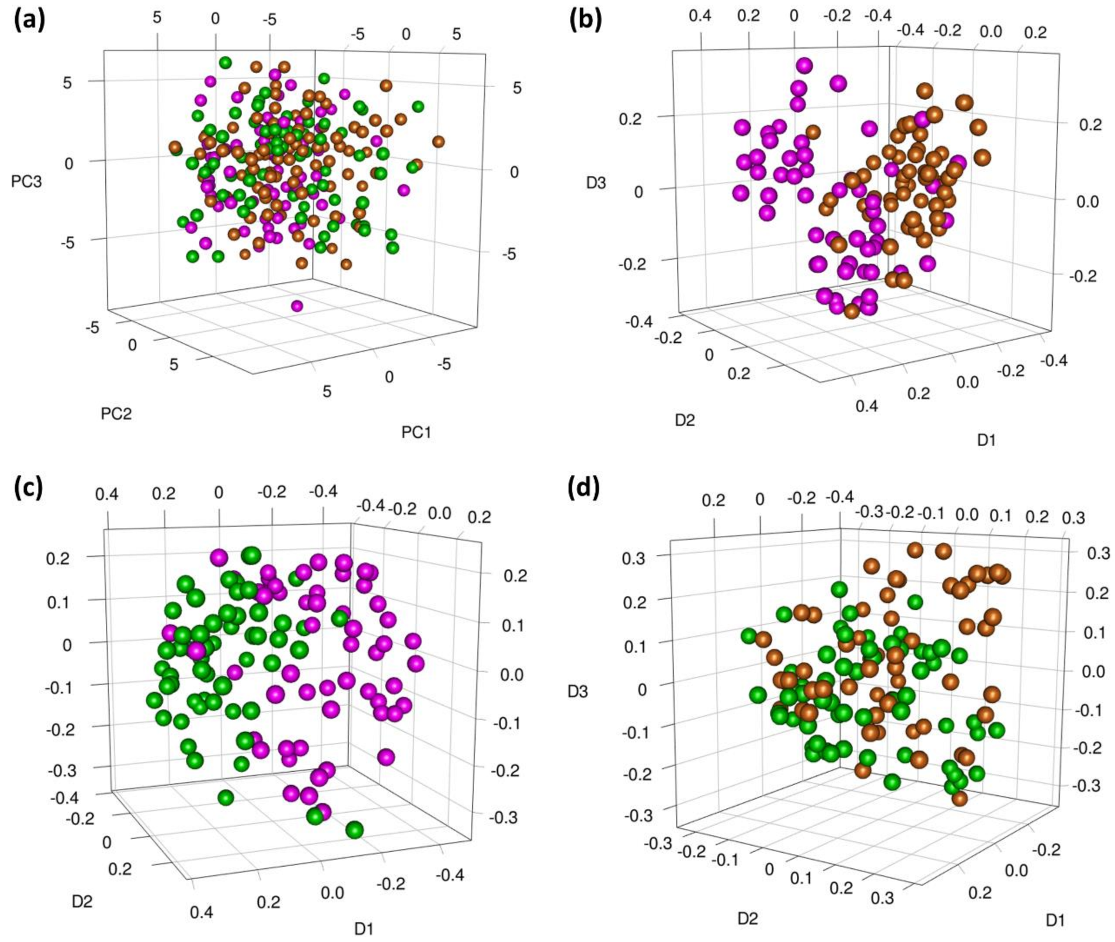

Figure S2. (a) Principal Component Analysis (PCA) model score plot (PC1 (13.4%) vs PC2 (8.0%) vs PC3 (7.3%)). Each dot represents a single metabolic profile colored by the different groups of patients: n=65 CRC patients (magenta dots), n=74 PP patients (green dots), and n=87 CTR patients (dark orange dots). Balanced Random Forest score plot of the models discriminating: (b) n=55 randomly selected CRC (magenta dots) and n=55 randomly selected CTR patients (dark orange dots); (c) n =55 randomly selected CRC (magenta dots) and n=55 randomly selected PP patients (green dots); (d) n=62 randomly selected PP (green dots) and n=62 randomly selected CTR patients (dark orange dots).

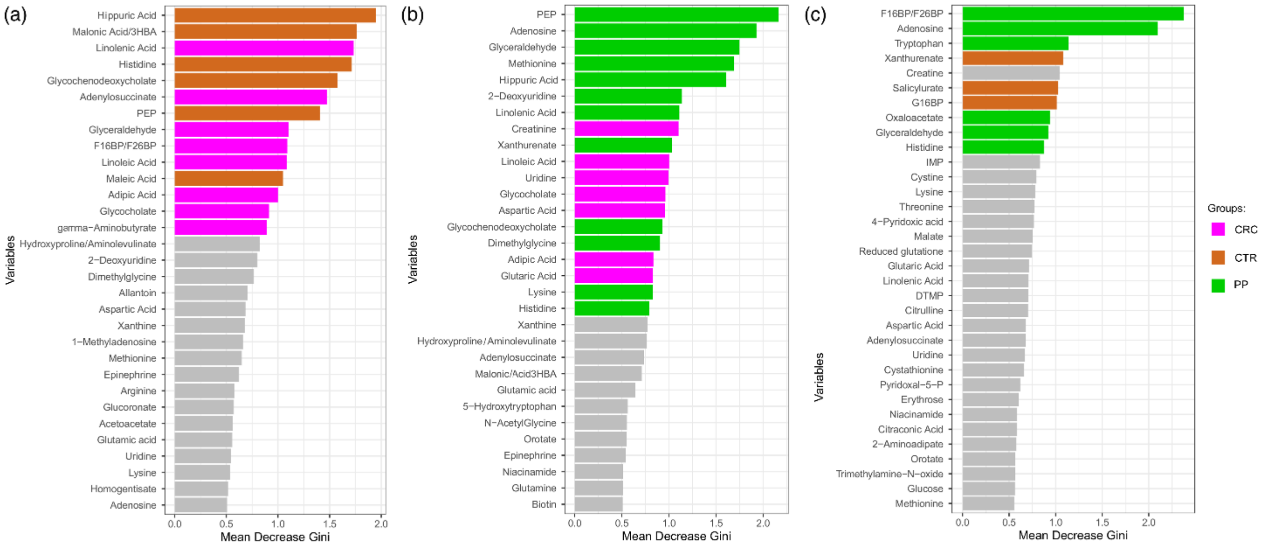

Figure S3: Metabolite importance calculated by Mean Decrease Gini score for classification between: (a) CRC and CTR, (b) PP and CTR, and (c) CRC and PP. Grey bars correspond to no significant variables. Magenta, green, and dark orange bars correspond, respectively, to statistically significant (FDR adjusted *P*-value < 0.05) and important variables more representative in CRC, PP, and CTR subjects. Only variables with a Mean Decrease Gini score > 0.5 was considered.

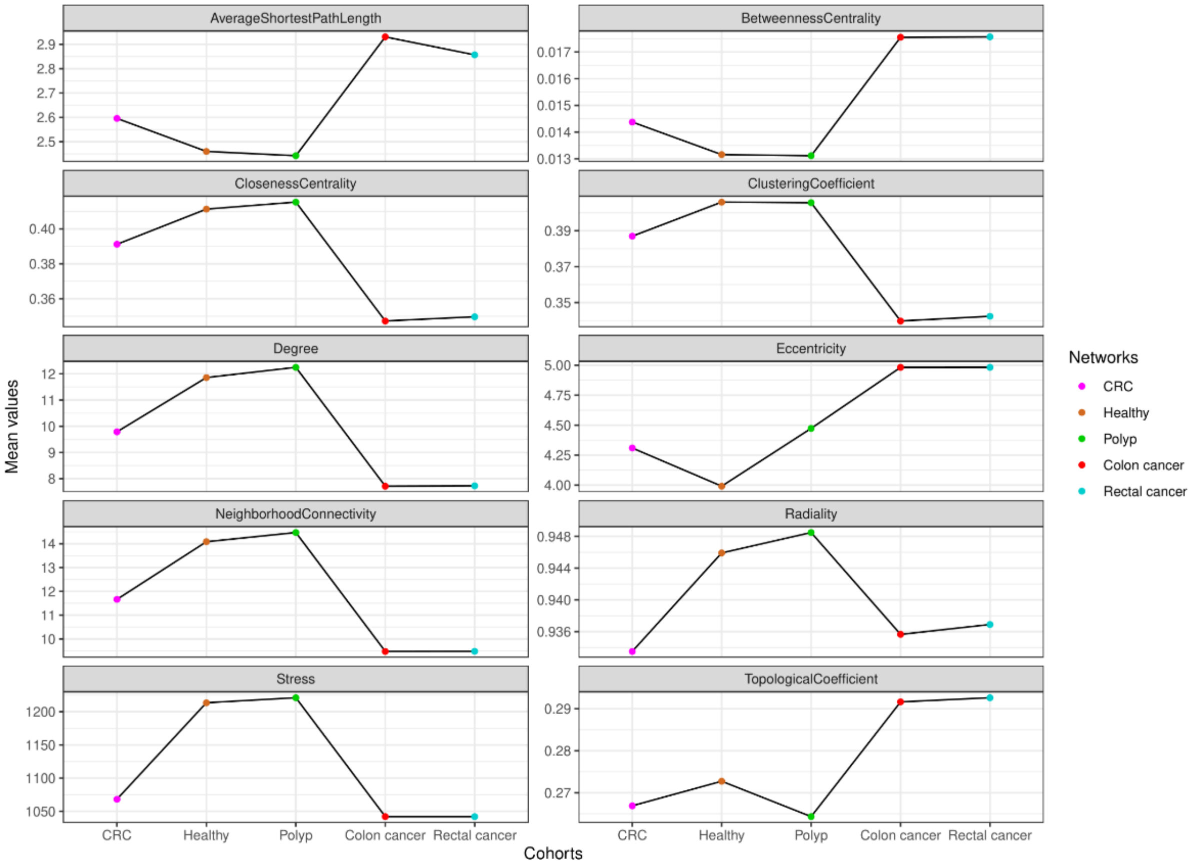

Figure S4: Topological network parameters of metabolite-metabolite association networks performed on CRC group (magenta dot), healthy control group (dark orange dot), polyposis group (green dot), colon cancer subject sub-group (red dot), and rectal cancer subject sub- group (light blue dot).
